# Cardiac profile of asymptomatic children with Becker and Duchenne muscular dystrophy under treatment with steroids and with/without perindopril

**DOI:** 10.1186/s12872-017-0627-x

**Published:** 2017-07-24

**Authors:** Sophie Mavrogeni, Aikaterini Giannakopoulou, Antigoni Papavasiliou, George Markousis-Mavrogenis, Roser Pons, Evangelos Karanasios, Michel Noutsias, Genovefa Kolovou, George Papadopoulos

**Affiliations:** 1 Onassis Cardiac Surgery Center, 50 Esperou Street, 175-61 P.Faliro, Athens, Greece; 2 Aghia Sophia Children’s Hospital, Athens, Greece; 3 Pentelis Children’s Hospital, Athens, Greece; 4 Department of Internal Medicine III, Division of Cardiology, Angiology and Intensive Medical Care, University Hospital Halle, Martin-Luther-University Halle, Ernst-Grube-Straße 40, D-06120 Halle (Saale), Germany

**Keywords:** Cardiovascular magnetic resonance, Duchenne/ Becker muscular dystrophy

## Abstract

**Background:**

To evaluate cardiovascular function in boys with Duchenne (DMD) and Becker (BMD) muscular dystrophy, using cardiac magnetic resonance (CMR).

**Methods:**

This is a single point cross sectional study of twenty-four boys with genetically ascertained DMD, and 10 with BMD, aged 10.5 ± 1.5 years (range 9–13), were prospectively evaluated by a 1.5 T system and compared with those of age-sex matched controls. The DMD patients were divided in 2 groups. Group A (*N* = 12) were under treatment with both deflazacort and perindopril, while Group B (*n* = 12) were under treatment with deflazacort, only. BMD patients did not take any medication. Biventricular function was assessed using a standard SSFP sequence. Late gadolinium enhancement (LGE) was assessed from T1 images taken 15 min after injection of 0.2 mg/Kg gadolinium DTPA using a 3D–T1-TFE sequence.

**Results:**

Group A and BMDs were asymptomatic with normal ECG, 24 h ECG recording and echocardiogram. Group B were asymptomatic but 6/12 had abnormal ECG and mildly impaired LVEF. Their 24 h ECG recording revealed supraventricular and ventricular extrasystoles (all at 12–13 yrs). LV indices in Group A and BMD did not differ from those of controls. However, LV indices in Group B were significantly impaired compared with controls, Group A and BMDs (*p* < 0.001). An epicardial LGE area = 3 ± 0.5% of LV mass was identified in the posterolateral wall of LV only in 6/12 patients of Group B, but in not in any BMD or Group A.

**Conclusion:**

Children with either BMD or DMD under treatment with both deflazacort and perindopril present preserved LV function and lack of LGE. However, further large scale multicenter studies are warranted to confirm these data, including further CMR mapping approaches.

## Background

Duchenne muscular dystrophy (DMD) is an X-linked recessive disorder affecting 1 in 3500 males [1, 2], due to mutations in dystrophin gene leading to reduced or deficient synthesis of dystrophin, a part of the dystrophin-glycoprotein complex (DGC) [3]. Dystrophin is expressed not only in skeletal but also in cardiac muscle and in brain. Therefore, although DMD is a primary degenerative condition of the skeletal muscles, it also affects seriously the heart [4].

DMDs become symptomatic before 5 years of age**,** present a loss of ambulation between ages 7 and 12 years**.** Cardiomyopathy and respiratory muscle dysfunction occur several years after the onset of neuromuscular symptoms and contribute to death usually in the 20s, with the chance of surviving to age 25–30, due to advances in respiratory care. The ECG is abnormal in 90% of cases, demonstrating tall R waves, increased RS in V1 and deep narrow Q waves in left precordial leads. Sinus tachycardia is the commonest abnormality. Atrial fibrillation, flutter and ventricular premature complexes can also be found. The main cause of death is heart failure; however, the clinical presentation of heart disease in DMD is masked by muscle weakness contributing to reduced physical activity [4]. Therefore, the early diagnosis is of great value in order to early treat preclinical heart disease.

Becker muscular dystrophy (BMD) is a milder form, also caused by mutations in dystrophin gene. BMDs have a more variable presentation of skeletal muscle weakness. They can usually walk and have a nearly normal life expectancy with most patients surviving to age 40–50. However, they have a 50% chance to develop cardiac involvement independently of the severity of skeletal muscle disease [5]. The ECG is abnormal in 75% of BMD, demonstrating tall R waves, increased RS in V1 and deep narrow Q waves in left precordial leads and or incomplete right bundle branch block. In both diseases elevated serum creatinine kinase activity is observed (over 5–10 fold normal values).

In both skeletal and cardiac muscle, the role of dystrophin is to protect from the contraction-induced damage. The lack or abnormal dystrophin, leads to cardio-myocytes’ death that can be accompanied by chest pain and diagnosed by the release of cardiac troponin into the serum [6]. After demise of cardiomyocytes, fibroblasts form a scar tissue leading in multifocal myocardial fibrosis (MF) [7, 8], starting from the epi- and progresses into the endocardium [9]. MF leads to ventricular wall thinning, loss of contractility and to dilated cardiomyopathy (DCM) [8], which has been described in both DMD patients [8] and carriers [10].

Late gadolinium enhancement (LGE), assessed by cardiac magnetic resonance (CMR), is the best non-invasive index for fibrosis detection and has been successfully used in the evaluation of both ischemic and non-ischemic cardiomyopathy [11, 12]. CMR has been also used to identify myocardial fibrosis during the early stages of cardiomyopathy in DMD/BMD [13–18] and its presence was associated with worse patient prognosis [16].

Preliminary data support the efficacy of angiotensin-converting enzyme inhibitors on left ventricular (LV) function. It was published the long-term impact of a preventive treatment with perindopril on mortality in children with DMD. However, this was a survival study, without providing tissue characterisation information. Additionally, the potential effect of co-administration of perindopril and deflazacort was not presented [19]. Additionaly, DMD patients under deflazacort were proven to have better cardiac parameters, measured by CMR, compared to those without deflazacort [20].

Our aim was to apply a CMR protocol, including evaluation of left ventricular function and LGE, in DMD patients during childhood under treatment either with both deflazacort and perindopril or only with deflazacort and compare them with BMD patients who were not under any kind of cardiac treatment and age-sex matched controls, in order to assess if the combination of deflazacort and perindopril is more beneficial than deflazacort monotherapy.

## Methods

### Patients

This is a single point cross sectional study of twenty-four boys with genetically ascertained DMD and 10 with BMD, aged 10.5 ± 1.5 years (range 9–13) have been prospectively evaluated both clinically and by CMR using a 1.5 T system and their results were compared with those of age-sex matched controls. The DMD population included 2 groups. Goup A (*N* = 12) under treatment with both deflazacort and perindopril and Group B (*n* = 12) under treatment only with deflazacort. Patients were included in Group A, if they tolerated a 1-mg test dose of perindopril; and if they had a systolic blood pressure ≥ 80 mmHg in the supine or >70 mmHg in the sitting position. Patients with a blood urea nitrogen >7 mmol/l, or contraindications to ACEI therapy, were included in Group B. BMD patients did not take any medication.

The diagnosis of BMD/DMD was documented by muscle biopsy demonstrating reduced levels of dystrophin in the immunohistochemical stainings and dystrophin analysis together with a genetic DMD locus study led to the diagnosis of BMD/DMD, with a gene deletion extending from exons between 31 to 62 for DMD and 45 to 55 for BMD. Additionally, all DMD patients presented all clinical characteristics of the disease since their early childwood.

Before the CMR evaluation, all DMD patients had a detailed clinical evaluation including personal and family history, physical examination, ECG, 24 h rhythm recording and echocardiogram. All DMD patients were under treatment with deflazacort (DFZ) (0.9 mg/kg/day, maximum daily dose: 36–39 mg) along with daily oral supplements of vitamin D and calcium starting at the age of 6 yrs. All DMD patients were monitored for potential adverse effects and had yearly ophthalmologic assessment for cataracts as well as examination of bone density and bone metabolism. Patients of Group A were also under treatment with perindopril 2 to 4 mg daily as tolerated starting at the age of 8 yrs. Both deflazacort and perindopril have been well tolerated by the DMD patients and treatment discontinuation was not needed in any of them. A written consent form was obtained from all parents, and the study was approved by the hospital’s ethics committee.

### Methods

The CMR evaluation was performed using a 1.5 T system and included functional and fibrosis evaluation.

#### CMR functional study.

For each subject, localizing scans were obtained to define the long (2-chamber) axis of the left ventricle. A mid ventricular short axis view was then prescribed, and used to plan a 4-chamber view. The short axis orientation was then defined accurately, perpendicular to both the 2- and 4-chamber views. To cover the entire left ventricle, 10 contiguous (gap = 0 mm) short axis slices were acquired in each study. The imaging sequence was a 2D, multi-phase (16 cardiac phases were acquired per cardiac cycle resulting to a temporal resolution of 47 ms for a heart rate of 80 beats/min), steady-state free-precession (SSFP) sequence (TE = 1.5 ms, TR = 3.1 ms, flip angle = 70°, slice thickness = 8 mm, acquired in-plane spatial resolution = 1.8 mm × 2.0 mm) characterized by the application of balanced gradients in all directions [10].

#### Fibrosis evaluation

To assess fibrosis 0.2 mmol/kg Gd-DTPA was given again and late gadolinium-enhanced images (LGE) were taken 15 min later, using a 3D–T1-TFE sequence, preconditioned with a 180 degrees inversion pulse (flip angle =15°, TE = 1.4 msec, TR = 5.5 ms, TI 225 to 275 ms as individually optimized to null myocardial signal, matrix 256X192 and slice thickness = 5 mm). Short axis, horizontal and vertical long axis images with gap = 0 covering the whole myocardium were taken in all patients [10].

#### Image analysis

To assess LGE, all short-axis slices from base to apex were inspected visually to identify areas of normal (completely nulled) myocardium. Mean signal intensity and standard deviation (SD) was derived and a threshold of >4 SD exceeding the mean was used to define areas of LGE. Summing the planimetered areas of LGE in all short- axis slices yielded the total volume, which was also expressed as a proportion of total LV myocardium (% LGE). The LGE analysis was performed by one experienced reader and reviewed and confirmed by a second expert reader with both of the independent readers blinded to patient’s identity and clinical profile. Any discrepancies in analysis between the 2 readers was then adjudicated by a senior reader with >10 yrs. of CMR experience, also blinded to patient’s identity and clinical profile.

Cine images were used for the evaluation of left ventricular ejection fraction (LVEF). Left ventricular endocardial borders were outlined on the end-systolic and end-diastolic short axis view images covering the entire LV. Papillary muscles were considered myocardium. LVEF was calculated as follows: LVEF = [(volume at end-diastole – volume at end-systole) / volume at end-diastole]. The MRI-MASS, Medis, Leiden, the Netherlands software was used and the readers were blinded to the clinical data (10).

#### Statistical analysis

All measurements were expressed as mean ± SD. Statistical significance of the differences was investigated using unpaired Student’s T-test. Correlation between variables was sought with Pearson’s correlation coefficient. For non-parametric data the Mann-Whitney test and Spearman’s correlation coefficient were used respectively. Statistical significance was considered for *p* < 0.05.

## Results

DMD patients had lower height, weight and body surface area compared to both BMD and controls (*p* < 0.05), but they did not differ in heart rate or blood pressure (Table 1). Deflazacort was started in all DMD patients at the age of 6 years and perindopril only in Group A at the age of 8 years, based upon drug tolerence.

**Table 1:** Baseline Characteristics of Study Groups

Characteristics	Group A (*n* = 12)	Group B (*n* = 12)	BMD Group (*n* = 10)	Controls (*n* = 34)
Age, yrs	10.5 ± 1.1	10.5 ± 1.3	10.9 ± 1.3	10.5 ± 1.5
Height, m	1.31 ± 0.03*	1.31 ± 0.04*	1.6 ± 0.04	1.62 ± 0.01
Weight, kg	37 ± 5.6*	39.7 ± 5*	58 ± 5.5	54.8 ± 2.6
BSA	1.16 ± 0.09*	1.22 ± 0.08*	1.6 ± 0.08	1.57 ± 0.04
Systolic blood pressure, mm Hg	106 ± 3	104 ± 5	101 ± 6	104 ± 3
Diastolic blood pressure, mm Hg	56 ± 4	55 ± 4	54 ± 4	57 ± 5
Heart rate, beats/min	92 ± 4	93 ± 4	96 ± 5	91 ± 6
Daily dose of perindopril, n				
2 mg	8	0	0	0
4 mg	4	0	0	0

*p < 0.05

Patients in Group A had no cardiac symptoms and had normal resting ECG, 24 h ECG recording and echocardiogram Patients in Group B had also no cardiac symptoms, but 6/12 had abnormal resting ECG and mildly impaired LVEF at the time of CMR evaluation. Since this was the first CMR evaluation of these patients and no previous data were available, the mildly impaired LV systolic function and late gadolinium enhancement of the myocardium) were seen in the same 6 patients concurrently. The 24 h ECG recording revealed rhythm disturbances, including sinus tachycardia, supraventricular and ventricular extrasystolic beats (all of them at the age of 12 years). No bradycardia, ventricular tachycardia and/or atrioventricular block were identified in any of them. BMD patients were asymptomatic, with normal resting ECG, 24 h ECG recording and echocardiogram.

**Fig. 1:**
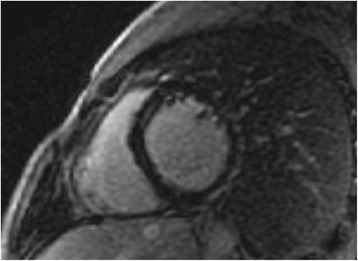
Inferolateral fibrosis in a patient with DMD under deflazacort, but without perindopril

LV indices in Group A and BMD did not differ from those of controls. However, LV indices in Group B were significantly impaired compared to controls, Group A and BMDs (*p* < 001). An epicardial LGE area = 3 ± 0.5% of LV mass was identified in the posterolateral wall of LV only in 6/12 patients of Group B (Fig. 1), but in none in any BMD patient or DMD from Group A. The detailed CMR data are presented in Table 2.

**Table 2:** CMR findings in DMD/BMD patients

Patients	LVESD (ml)	LVESV (ml)	LVEF (%)	LGE (% LV mass)
BMD	126.7 ± 4.7	42.7 ± 1.7	69.7 ± 1.33	0
DMD-Gr A	127.6 ± 3.7	43.083 ± 2.3	69.16 ± 1.6	0
DMD-Gr B	134.4 ± 8.17*	54.08 ± 4.27*	56.08 ± 3.5*	3 ± 0.5*
Controls	126.59 ± 5.59	41.9 ± 2.3	69.4 ± 1.3	0

*LVEDV* left ventricular end-diastolic volume, *LVESV* left ventricular end-systolic volume, *LVEF* left ventricular ejection fraction, *LGE* late gadolinium enhanced area

*P < 0.05

## Discussion

Our study showed that LV subclinical dysfunction and LGE can be found during childhood in DMDs but not in BMDs. These abnormalities represent early findings in DMDs under treatment with deflazacort, but not in BMDs or in DMDs under treatment with both deflazacort and perindopril and can be missed by the routine echocardiographic assessment.

We should notice that mild LV dysfunction, assessed by CMR, was an early finding in our Group B patients, despite the normal echocardiographic report. This is in agreement with previous studies supporting that echocardiographic assessment of LV has a weak correlation with CMR indices in children with DMD and remains suboptimal for clinical management [17]. Therefore, we propose that CMR should be performed routinely in children with DMD, not only for LGE imaging, but also for ventricular function assessment [17, 20–25].

In our study, LGE was also identified only in the older DMDs, who were not under treatment with perindopril. According to previous studies, LGE represents a sensitive index for the early diagnosis of cardiomyopathy in DMD/BMD carriers and patients and is a marker of worse prognosis [10, 14–25]. Another study documented that progressive myocardial fibrosis, as detected by LGE, was strongly correlated with the LVEF decline in DMD; the longer steroid treatment duration was associated with a lower age-related increase in myocardial fibrosis burden [24]. Our results were in agreement with these studies identifying LGE in the eldest DMDs with mildly impaired LVEF. The lack of LGE in young BMDs was also in agreement with the literature supporting the late development of cardiomyopathy in BMD [5].

We may hypothesize parallels between myocarditis and its sequelae inflammatory cardiomyopathy (DCMi), which is also characterized by CMR detectable LGE, that is significantly associated with the immunohistologically detected intramyocardial inflammation [25]. However, in DMD, the initiating mechanism of this anticardiac inflammatory cascade is the inherited cardiomyopathy, and not the acquired cardiotropic viral infection which triggers most cases of myocarditis [26, 27].

Recently, it was documented that also LGE-spared regions of boys with DMD have significantly different native T1 and ECV values compared to controls and native T1 measurements can identify early changes in DMD patients without the presence of LGE and predict disease severity more effectively than ECV [28]. However, in our patients T1 and ECV values were not available.

Although some of DMD patients presented early ECG changes and ECG is a cheap and widely available tool, the absence of ECG changes in DMD can not exclude the cardiac involvement [13]. Furthermore, a recent study showed that although there was a positive trend of correlation between fragmented QRS and the amount of myocardial fibrosis as assessed by LGE, the statistical significance of the relationship was low [29].

In this context, CMR seems to be the only objective way to early detect asymptomatic myocardial involvement in dystrophinopathies. However, in early childhood a CMR evaluation may be problematic, because it may need sedation and there may be long term issues with repeated use of gadolinium. However, the recent application of native T1 mapping gives more reliable information than LGE with the additional advantage of not using contrast agent [28].

Another important point is the effect of steroids in DMDs. A recent study evaluating the effects of steroid use alone or in combination with angiotensin converting enzyme inhibitors (ACEI) or angiotensin receptor blocker (ARB) using cardiovascular magnetic resonance (CMR) derived circumferential strain did not document any significant difference [29]. However, previous studies by Duboc et al. documented that the early initiation of treatment with perindopril in DMD patients, aged 9.5 and 13 years, presenting with normal LV ejection fraction, was associated with a lower mortality [30]. Our study also proved that DMDs under treatment with both deflazacort and perindopril were significantly benefited compared to those treated only with deflazacort. Our study in comparison with the previous study by Hor KN et al. [31] has the additional advantage of being focused not only on functional information but also on a robust index such as LGE, which was not provided by Hor KN et al. and is currently considered as the trademark lesion of dystrophinopathies [23]. However, our small population study did not allow clear conclusions.

### Limitations of the study

The current study has the limitation of a relatively small population sample. We also did not pursue either a short or a long term follow up. Additionally, T1 mapping measurements, which could potentially reveal additional abnormalities as compared to LGE alone, were not available.

Finally, the study design can not definitely support that perindopril is protective in DMD, because the selection of patients with and without perindopril was not random, but subject to tolerance. Also, as a cross-sectional study, it can only show association, and neither causation nor progression. However, we strongly believe that if our study will be published, it can motivate further longitudinal cohort studies that may show more supportive results.

## Conclusion

Children with either BMD or DMD under treatment with both deflazacort and perindopril present preserved LV function and lack of LGE. However, further large scale multicenter studies are warranted to confirm these data, including further CMR mapping approaches.

## Abbreviations

ACEI: angiotensin converting enzyme inhibitors
ARB: angiotensin receptor blocker
BMD: Becker muscular dystrophy
CMR: Cardiovascular magnetic resonance
DGC: dystrophin-glycoprotein complex
DMD: Duchenne muscular dystrophy
ECG: Electrocardiogram
ECV: Extracellular volume fraction
LGE: Late gadolinium enhancement
LVEF: Left ventricular ejection fraction
SSFP: steady-state free-precession sequence
3D: T1-TFE sequence
TE: Echo time
TR: Repetition time
TI: Inversion recovery time
